# Modulation of growth, gut microbiota, and health markers in rabbits fed hydroponic barley with or without enzymes

**DOI:** 10.3389/fvets.2025.1615198

**Published:** 2025-07-18

**Authors:** Ahmed A. A. Abdel-Wareth, Esraa M. H. Mohamed, Hamdy A. Hassan, Rokia B. Elamary, Zainab Al-Amgad, Jayant Lohakare

**Affiliations:** ^1^Department of Animal and Poultry Production, Faculty of Agriculture, South Valley University, Qena, Egypt; ^2^Poultry Center, Cooperative Agricultural Research Center, Prairie View A&M University, Prairie View, TX, United States; ^3^Department of Botany and Microbiology, Faculty of Science, Luxor University, Luxor, Egypt; ^4^General Authority for Veterinary Services, Qena Veterinary Directorate, Qena, Egypt

**Keywords:** hydroponic barley, microbiota, histology, physiology, enzyme, rabbit

## Abstract

**Introduction:**

Hydroponic barley (HB) has emerged as a sustainable alternative feed ingredient; however, its effects on rabbit physiology and gut health remain underexplored. This study aimed to evaluate the impact of dietary HB, with or without enzyme supplementation, on the growth performance, cecal microbiota, and organ histology of growing rabbits.

**Methods:**

Sixty male Hy-Plus rabbits were randomly allocated to one of three dietary groups: a control group, a group receiving 25% hydroponic barley (CHB), and a group receiving 25% hydroponic barley supplemented with 0.5 g/kg of AXTRA XB enzymes (CHBE). The feeding trial lasted for 60 days. Growth performance parameters, serum biochemistry, cecal microbiota, and histological features of the liver and intestines were evaluated. Data were analyzed using one-way ANOVA.

**Results:**

Rabbits fed the CHB and CHBE diets showed significant improvements in body weight, weight gain, and feed conversion ratio compared to the control group. Both treatment groups exhibited beneficial modulation of cecal microbiota, with increased Lactobacillus spp. and reduced Escherichia coli populations. Serum biochemical profiles were improved, as evidenced by lower levels of aspartate aminotransferase, alanine aminotransferase, urea, creatinine, and cholesterol. Histological examination revealed normal liver and intestinal structures in all treatment groups.

**Discussion:**

Incorporating 25% hydroponic barley, with or without enzyme supplementation, improved growth performance, enhanced gut microbiota composition, and supported organ health in growing rabbits. These findings support the potential of hydroponic barley as a functional and sustainable feed ingredient in rabbit production.

## 1 Introduction

Rabbit farming is gaining attention as an environmentally sustainable alternative to traditional livestock production, thanks to rabbits’ rapid reproduction and low environmental impact (1, 2). As global sustainability concerns rise, rabbit meat, a low-fat, high-quality protein, is becoming an appealing option for health-conscious consumers (2). This shift is expected to increase demand for rabbit meat. To improve rabbit health and productivity, it’s essential to provide a balanced diet that supports gut health. Regular monitoring and tailored feeding strategies are crucial for optimal growth and well-being (2). Since feed costs make up over 70% of production expenses, cost-effective feeding strategies are key to ensuring both economic growth and sustainability (3). In response to the limitations of traditional green fodder cultivation, hydroponics has emerged as an innovative and sustainable method for growing rabbit feed (4). This soil-free cultivation technique offers an efficient solution to meet the rising nutritional demands for rabbit feed while significantly reducing reliance on land and water resources. Hydroponics enables the rapid, high-volume production of nutrient-dense fodder in a cost-effective and environmentally friendly manner (5–8).

Hydroponic barley (HB) has shown promising nutritional benefits when included in rabbit diets. Studies have demonstrated that incorporating HB at levels of 20–40% improves serum metabolic profiles and carcass characteristics in rabbits (9, 10). Furthermore, rabbits fed HB diets exhibit increased viable bacterial counts in the cecum, contributing to improved gut health (11). Previous research from our group has also shown that replacing 25% of a conventional diet with hydroponic, whether supplemented with enzymes or not, results in enhanced growth performance, improved nutritional digestibility, and greater economic efficiency in growing rabbits (12).

Enzyme supplementation plays a crucial role in enhancing fiber digestibility, which leads to improved nutrient absorption and overall utilization (13). This is particularly important for hydroponic barley, which is naturally high in fiber, a component that can limit its nutritional value if not properly broken down. Additionally, enzymes can support gut health by reducing the viscosity of digesta, which in turn enhances metabolic efficiency (14). Specifically, xylanase and beta-glucanase were chosen due to their ability to break down complex carbohydrates, such as xylans and beta-glucans, that are abundant in the cell walls of hydroponic barley (15). Xylanase targets hemicellulose, a major fiber component, while beta-glucanase acts on the beta-glucan fraction (13–15). By facilitating the degradation of these complex fibers, these enzymes improve the digestibility and nutritional value of hydroponic barley, leading to enhanced nutrient absorption and potentially improving the growth performance and overall health of rabbits. Despite these encouraging findings, there remains limited research investigating the potential of enzyme supplementation in hydroponic barley diets to enhance further gut function, microbiota composition, and overall growth performance in rabbits. Therefore, assessing how these diets influence various physiological aspects fundamental to rabbit health and productivity is crucial. Understanding the impact of these diets on gut health plays a key role in digestion, immunity, and overall health, which may influence growth performance and disease resistance (16, 17). Additionally, examining carcass characteristics and histological responses provides insights into how these dietary changes affect the quality and yield of rabbit meat, offering valuable information for improving production efficiency. Finally, evaluating physiological responses helps determine the broader effects of these dietary interventions on rabbit health, including metabolic and immune function. Thus, this study aims to evaluate the nutritive value of HB by substituting 25% of a control diet with hydroponic barley, with or without enzyme supplementation. Furthermore, we will assess the effects of these diets on production, cecal microbiota, carcass characteristics, and histological and physiological responses in growing rabbits.

## 2 Materials and methods

### 2.1 Experimental animals, design, and management

This study was carried out at the Rabbit Research Farm, Animal and Poultry Production Department, Agriculture Faculty, South Valley University, Qena, Egypt. The experimental protocol (Protocol No. SVUAGR8/2018) was approved by the Ethics Committee of the Animal and Poultry Production Department at South Valley University, and all procedures complied with the ARRIVE guidelines (18).

Sixty male Hy-plus rabbits, with an average body weight (BW) of 669 ± 12 g, were weaned at 30 days of age and housed individually in galvanized wire net cages (50 cm width, 60 cm length, 40 cm height). Each cage was fitted with a manual feeder and waterer, and all rabbits were housed in a closed system maintained at a consistent temperature of 22°C, with a 16-h light cycle and an 8-h dark cycle. The cages were equipped with stainless steel nipples for free access to fresh tap water. Throughout the experiment period (60 days), the health and physical condition of the rabbits were closely monitored.

The rabbits were randomly allocated into three experimental groups, each containing 20 rabbits. The first group was given a control diet (commercial rabbit diet) formulated to meet the nutritional needs (Table 1) of growing rabbits according to the recommendations of De Blas and Mateos (19). The second group was fed the control diet with 25% substitution of the control diet with dry hydroponic barley (CHB), while the third group received the CHB diet supplemented with 0.5 g/kg of enzymes (CHBE). The experiment lasted for 60 days. The diets were fed in pelleted form. The enzymes used (Xylanase and Beta Glucanase, AXTRA XB, Danisco Animal Nutrition, Marlborough, Wilts, UK) were included to assist in the digestion of the high-fiber diet. The endo-1,4-*β*-xylanase (12,200 U/g) is produced by a genetically modified strain of *Trichoderma reesei*, while the endo-1,3(4)-β-glucanase (1,520 U/g) is produced by a non-genetically modified strain of *T. reesei* (12). The supplemental enzymes (xylanase and β-glucanase; AXTRA® XB) are thermostable formulations with documented activity retention (>85%) after pelleting at 90°C (12). While post-pelleting assays were not performed here, their functional efficacy is evidenced by significant treatment effects on growth and gut morphology.

**Table 1:** Composition of the ingredients (as-fed basis) in the diet provided to rabbits during the 60-day study.

Ingredient	Percentage (%)
Corn	31.00
Wheat bran	20.00
Soybean meal (44% CP)	19.00
Wheat straw	12.00
Lucerne hay	5.00
Rice bran	5.00
Linseed straw	2.80
Sunflower meal	2.50
Limestone	2.00
Sodium chloride	0.30
Vitamin-mineral premix	0.30
DL-methionine	0.10
Total	100

^1^Per kg of diet: vitamin A 10,000 IU, vitamin D3 900 IU, vitamin E 50.0 mg, vitamin K 2.0 mg, vitamin B1 2.0 mg, folic acid 5.0 mg, pantothenic acid 20.0 mg, vitamin B6 2.0 mg, choline 1,200 mg, vitamin B12 0.01 mg, niacin 50 mg, biotin 0.2 mg, Cu 0.1 mg, Fe 75.0 mg, Mn 8.5 mg, Zn 70 mg.

### 2.2 Hydroponic barley production system

The hydroponic system used to grow barley was installed in a growth chamber at the Rabbit Research Farm, South Valley University, Qena, Egypt. The system included two metal-framed units (55 × 200 × 240, cm), each featuring four shelves and 24 planting trays. For barley seed cultivation, plastic trays measuring 90 × 30 × 4 cm were utilized. The system was kept at a consistent temperature of 24 ± 2°C and a relative humidity of 65–70%. Barley seeds (*Hordeum vulgare* L.) were obtained from the Department of Crops, Faculty of Agriculture, South Valley University. The seeds were first sterilized by soaking in a 20% sodium hypochlorite solution for 30 min, then rinsed, and soaked overnight in tap water. The seeds were planted in the trays, which were irrigated twice daily using tap water. On day 8, the barley fodder was harvested, divided by hand, and sun-dried before being incorporated into the rabbit diets. The diets and hydroponic barley samples were ground using a centrifugal mill to pass through a 1 mm screen. Subsequently, the diet samples were chemically analyzed (Table 2), including moisture content using oven drying (method 930.15), ash content by incineration (method 942.05), protein by the Kjeldahl method (method 984.13), and ether extract by Soxhlet fat analysis (method 954.02), as described by AOAC International (20).

**Table 2:** Chemical analysis of experimental diets and hydroponic barley.

Items %	Diets			
	Control	CHB	CHBE	HB
Dry matter	96.11	95.75	96.07	16.87
Organic matter	89.51	89.61	89.06	96.04
Ash	10.49	10.39	10.94	9.96
Crude protein	18.30	18.10	18.10	17.84
Crude fiber	12.30	13.30	12.80	12.30
Crude fat	4.33	4.40	4.70	4.36
Digestible energy (MJ/kg DM)^a^	9.35	9.47	9.45	9.92

Control: Basal diet.

CHB: 25% hydroponic barley replacement.

CHBE: 25% hydroponic barley +0.5 g/kg enzymes (AXTRA XB).

HB: Hydroponic barley.

a DE estimated according to Fekete (45).

### 2.3 Productive performance

Rabbit body weight (BW) was measured on days 30 and 90 of life. Feed intake for each cage was assessed by measuring the feed residue on the same day as the BW recordings during the whole period of the experiment. The feed conversion ratio was determined by dividing the amount of feed consumed by the corresponding BW gain, calculated based on the average weight gain per cage during the whole period of the experiment. Additionally, mortality was monitored daily throughout the entire experimental period of 60 days.

### 2.4 Clinical chemistry analysis

At 86 days of age, twenty rabbits from each treatment group were anesthetized with an intraperitoneal injection of sodium pentobarbital (60 mg/kg body weight). Blood was then collected from the ear vein using a sterile syringe and needle. The blood samples were immediately placed in serum collection tubes and allowed to clot at room temperature for 30 min. Following clotting, the samples were centrifuged at 3,000 rpm for 10 min to separate the serum from the blood cells. The serum was carefully transferred into clean tubes and stored at −20°C until analysis. This procedure ensures the preservation of serum for accurate biochemical analysis. The liver enzymes [aspartate aminotransferase (AST) and alanine aminotransferase (ALT)], as well as the total cholesterol, creatinine, and urea levels, were measured using commercial reagent kits sourced from Spectrum Chemical Company (Obour City, Cairo, Egypt). The analysis of these parameters was performed using a spectrophotometer, following the manufacturer’s instructions.

### 2.5 Carcass measurements

At the end of the experiment (90 days of age), all twenty rabbits from each treatment group were weighed and humanely slaughtered. After slaughter, the rabbits were bled, and various body parts, including the skin, genitalia, urinary bladder, gastrointestinal tract, and lower limbs, were excised. The carcass yield was then calculated by determining the dressing percentage, which is the proportion of hot carcass weight to BW, expressed as a percentage. The hot carcass, fore, middle (loin), and hind sections were weighed, and the weights of internal organs (liver, heart, lungs, kidneys, spleen, and testes) were measured and expressed as a percentage of the hot carcass weight.

### 2.6 Microbial enumeration

For determination of colony forming units, 1 g of each cecum sample from slaughtered animals (90 days of age) was aseptically immersed in selenite cysteine broth (SCB) medium (Oxoid®) and Tryptic soy broth as enrichment broth for *E. coli, Salmonella* sp., and *Lactobacillus* spp. respectively, and incubated at 37°C for 24 h. Fifteen-fold serial dilutions were made up and 0.1 mL of each dilution was spread using glass beads on eosin methylene blue (Oxoid®), *Salmonella Shigella* (Oxoid®) and MRS agar plates as selective media for *E. coli, Salmonella*, and *Lactobacillus* spp. respectively. Plates were incubated at 37°C for 24 h. Typical *E. coli*, Salmonella sp., and lactobacillus spp. colonies were counted only for plates with 30–300 colonies (21). The CFU number was then calculated per ml sample according to the following equation:

CFU/ml=No.of colonies×1/dilution

### 2.7 Histological examination of the small intestine

At the end of the experiment (90 days of age), the ileum (the last section of the small intestine) and the liver were harvested for histological analysis. Samples were preserved in 10% neutral buffered formalin, dehydrated through increasing concentrations of ethanol (75–100%), and embedded in paraffin. Sections (5 μm thick) were cut using a microtome (Leica RM 2155, England), stained with Hematoxylin and Eosin (H&E), and examined under a microscope (AmScope 5.0 MP) at 100× and 400× magnification. A total of 20 images per group were analyzed for histological monitoring.

### 2.8 Statistical analysis

The data were evaluated using a one-way analysis of variance (ANOVA) with the general linear model approach, as outlined in the SAS/STAT® 9.2 User’s Guide (SAS Institute, 22). The analysis model was structured to examine the fixed effects of the treatments and account for random errors in the data. All samples were processed and analyzed individually (*n* = 20 biologically independent replicates per treatment group). No pooling of samples was performed, ensuring each measurement represents a distinct experimental unit. Differences among treatment means were determined using Duncan’s multiple-range test (23). Statistical significance was accepted for *p*-values below 0.05, with values <0.001 reported as “0.001.”

## 3 Results

### 3.1 Growth performance

Table 3 presents the body weight, body weight gain, feed intake, and feed conversion ratio of growing rabbits aged 30–90 days. The body weight of rabbits in the CHB group was 11.3% higher, and the body weight in the CHBE group was 16.8% higher compared to the control group. Specifically, the CHB group had a body weight that was 11.3% greater than the control group, while the CHBE group exhibited a 16.8% increase in body weight over the control group. The body weight gain was also significantly greater in the CHB and CHBE groups. The CHB group showed an 18.5% increase in body weight gain compared to the control group, while the CHBE group experienced a 24% higher body weight gain than the control group. There were no significant differences in feed intake among the groups. The CHB group consumed 4.4% more feed than the control group, and the CHBE group consumed 5.2% more feed than the control. Both the CHB and CHBE groups demonstrated improved feed conversion ratios. The CHB group had a 12% improvement in feed conversion ratio compared to the control group, while the CHBE group showed a 15% improvement over the control group. These findings suggest that including hydroponic barley, especially when combined with enzyme supplementation, significantly improved growth performance and feed efficiency in rabbits. Both the CHB and CHBE groups showed marked improvements in body weight, body weight gain, and feed conversion ratio compared to the control group.

**Table 3:** Effects of dietary hydroponic barley (HB) with or without enzyme supplementation on growth performance of growing rabbits.

Items	Treatments			SEM	*p*-value
	Control	CHB	CHBE		
Initial body weight, g	668	652	689	16	0.649
Final body weight, g	1946^b^	2166^a^	2273^a^	39	0.001
Total body weight gain, g	1278^b^	1514^a^	1584^a^	37	0.001
Total feed intake, g	4,974	5,191	5,231	64	0.222
Feed conversion ratio	3.917^a^	3.446^b^	3.322^b^	0.084	0.004

Values (*n* = 20) in the same row with different superscripts (^a,b^) are significantly different (*p* < 0.05).

SEM: Standard error of the means.

Control: Basal diet.

CHB: 25% hydroponic barley replacement.

CHBE: 25% hydroponic barley +0.5 g/kg enzymes (AXTRA XB).

### 3.2 Blood biochemical constituents of rabbits

**Figure 1:**
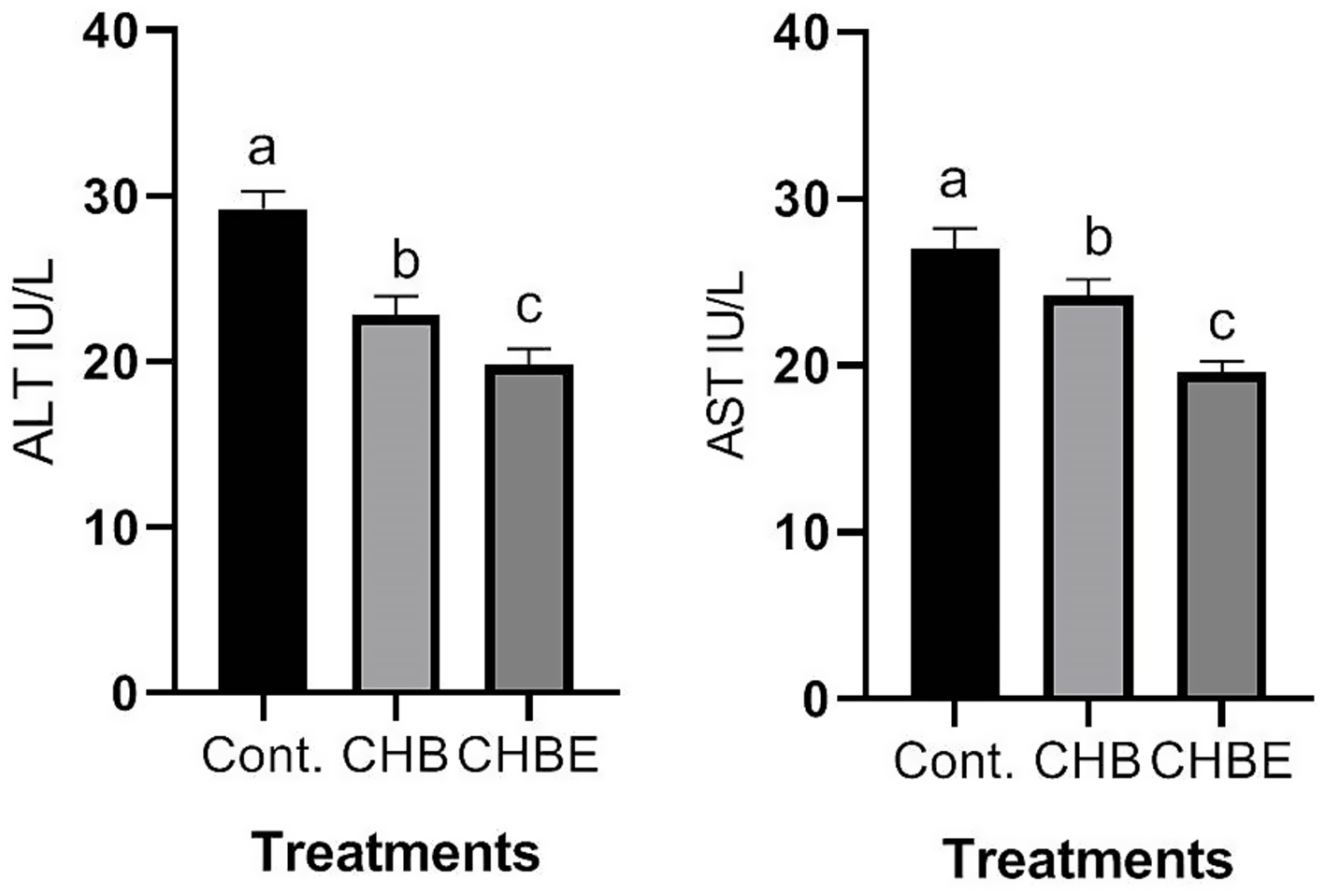
Liver enzyme activity in male rabbits at 86 days of age following control (Basal diet), 25% hydroponic barley replacement (CHB), and 25% hydroponic barley +0.5 g/kg enzymes (CHBE) diets. Statistical analysis was performed to identify significant differences between groups, with mean comparisons conducted using Duncan’s multiple range test (*n* = 20). Values within the same scale bar with different superscripts (a–c) are significantly different (*p* < 0.05).

**Figure 2:**
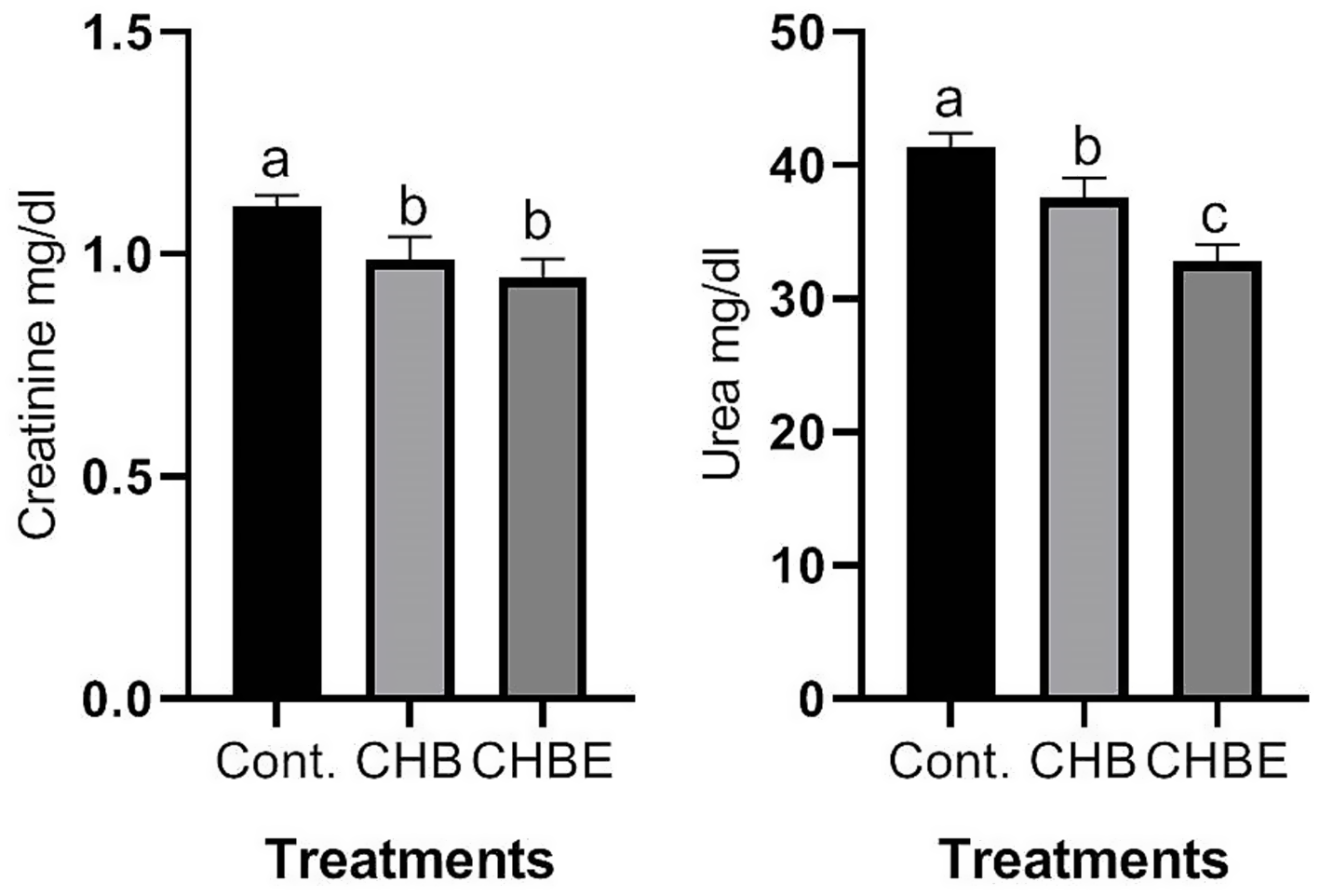
The kidney function of male rabbits in response to control (Basal diet), 25% hydroponic barley replacement (CHB), and 25% hydroponic barley +0.5 g/kg enzymes (CHBE) diets at 86 days of age. Statistical analysis was performed to identify significant differences between groups, with mean comparisons conducted using Duncan’s multiple range test (*n* = 20). Values within the same scale bar with different superscripts (a–c) are significantly different (*p* < 0.05).

**Figure 3:**
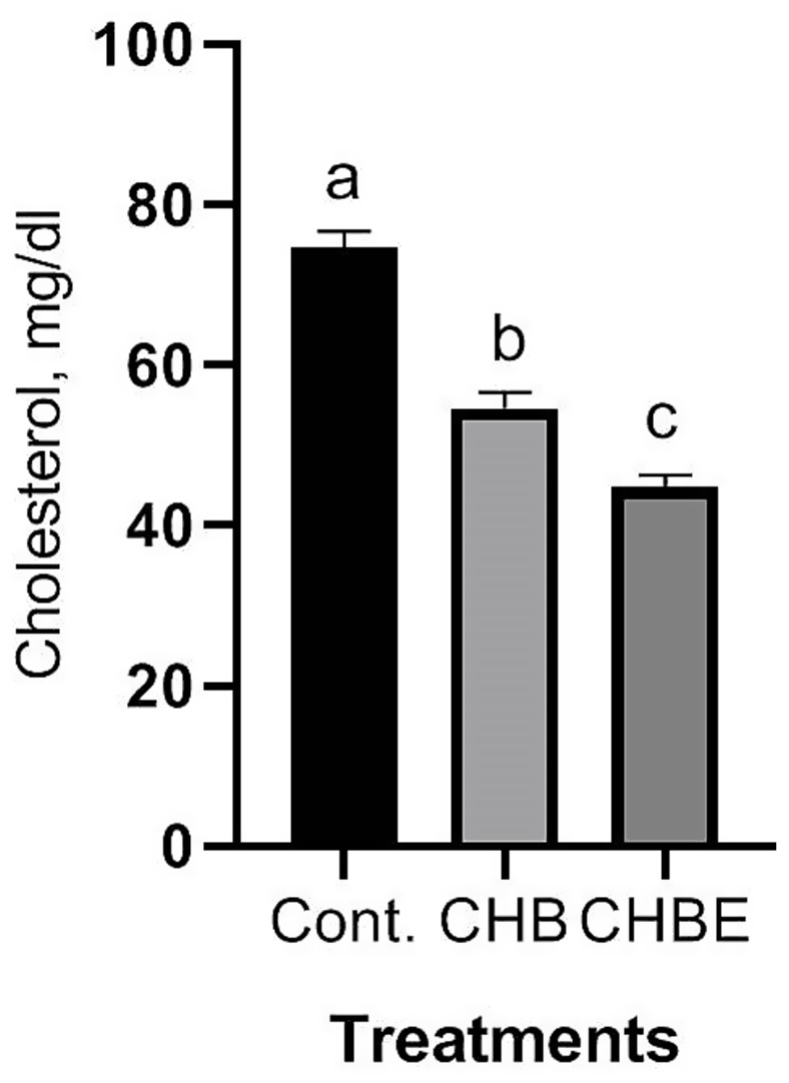
Cholesterol (mg/dl) of male rabbits in response to control (Basal diet), 25% hydroponic barley replacement (CHB), and 25% hydroponic barley +0.5 g/kg enzymes (CHBE) diets at 86 days of age. Statistical analysis was performed to identify significant differences between groups, with mean comparisons conducted using Duncan’s multiple range test (*n* = 20). Values within the same scale bar with different superscripts (a–c) are significantly different (*p* < 0.05).

Figures 1–3 illustrate the effects of hydroponic barley, with or without enzyme supplementation, on the serum levels of ALT, AST, urea, creatinine, and cholesterol in growing rabbits. Rabbits fed CHB and CHBE diets showed a significant reduction (*p* < 0.05) in serum ALT and AST activity compared to the control group (Figure 1). Similarly, blood urea and creatinine levels were significantly lower (*p* < 0.05) in rabbits on CHB and CHBE diets compared to the control (Figure 2). Furthermore, serum cholesterol levels were significantly reduced (*p* < 0.05) in the rabbits fed CHB and CHBE diets compared to the control group (Figure 3).

### 3.3 Carcass criteria and internal organs

The carcass characteristics of rabbits fed diets substituted with 25% hydroponic barley (HB), with or without enzyme supplementation, are presented in Table 4. Rabbits on the CHB and CHBE diets showed a significant increase in dressing percentage and a reduction in the fat-to-BW ratio (*p* < 0.05) compared to the control group. Additionally, cecum length was significantly greater in rabbits fed CHB and CHBE diets compared to the control. However, there were no significant differences in the weight of the brain, liver, heart, spleen, kidneys, intestine, or other organs between the treatment groups.

**Table 4:** Effects of dietary hydroponic barley (HB) with or without enzyme supplementation on carcass characteristics of growing rabbits.

Items	Treatments			SEM	*p*-value
	Control	CHB	CHBE		
Live body weight, g	2072^b^	2191^ab^	2295^a^	37.3	0.040
Dressing%	52.67^b^	54.60^ab^	55.71^a^	0.511	0.036
Fat, %	0.902^a^	0.616^b^	0.642^b^	0.055	0.054
Liver, %	2.940	3.505	3.534	0.152	0.206
Heart, %	0.311	0.296	0.314	0.012	0.818
Kidney, %	0.836	0.760	0.838	0.023	0.309
Spleen, %	0.074	0.062	0.054	0.007	0.497
Intestine, %	18.35	15.17	13.69	0.975	0.135
Cecum, %	0.496	0.554	0.606	0.043	0.602
Length of the intestine, cm	286	324	337	13.2	0.270
Cecum length, cm	10.00^b^	11.83^ab^	12.67^a^	0.470	0.046
Lungs, %	0.905	0.939	0.916	0.041	0.950
Head, %	4.807	4.652	4.886	0.101	0.660

Values (*n* = 20) in the same row with different superscripts (^a,b^) are significantly different (*p* < 0.05).

SEM: Standard error of the means.

Control: Basal diet.

CHB: 25% hydroponic barley replacement.

CHBE: 25% hydroponic barley +0.5 g/kg enzymes (AXTRA XB).

### 3.4 Microbial populations

Table 5 shows the microbial populations in the cecal of rabbits. Rabbits fed CHB and CHBE had a significant increase (*p* < 0.004) in cecal *Lactobacillus* spp. and a significant decrease (*p* < 0.001) in *E. coli* compared with the control group. On the other hand, *Salmonella* was absent in rabbits fed CHB and CHBE compared with the control group.

**Table 5:** Effects of dietary hydroponic barley (HB) with or without enzyme supplementation on cecal microbial populations (log_10_ CFU/g) in growing rabbits.

Items	Treatments			SEM	*p*-value
	Control	CHB	CHBE		
*E. coli*, (log_10_ cfu/g)	13.30^a^	11.40^b^	7.11^c^	1.10	0.001
*Salmonella* sp. (log_10_ cfu/g)	1.11	absent	absent	000	000
*Lactobacillus* sp. (log_10_ cfu/g)	8.12^c^	14.47^a^	12.30^b^	0.055	0.004

Values (*n* = 20) in the same row with different superscripts (^a,b^) are significantly different (*p* < 0.05).

SEM: Standard error of the means.

Control: Basal diet.

CHB: 25% hydroponic barley replacement.

CHBE: 25% hydroponic barley +0.5 g/kg enzymes (AXTRA XB).

### 3.5 Histological response of rabbits

**Figure 4:**
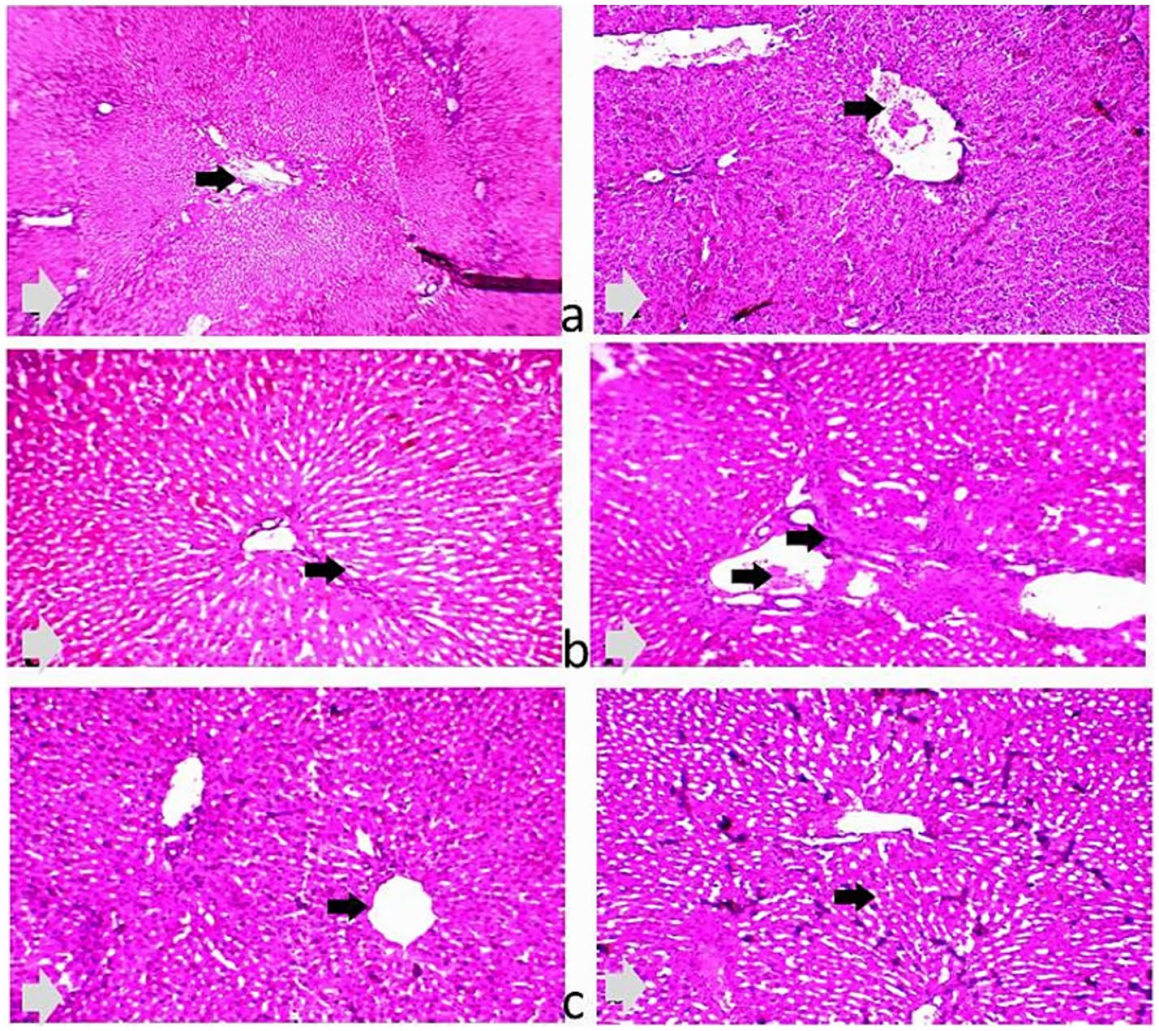
**(a–c)** Photomicrographs of the liver in the 25% hydroponic barley replacement (CHB), and 25% hydroponic barley +0.5 g/kg enzymes (CHBE) diets groups showing normally arranged hepatocytes and intact blood vessels **(a,b)**. In contrast, the liver of the control group **(c)** exhibited slight congestion and mild dilation of the blood vessels. 40× and 100× (H&E staining).

**Figure 5:**
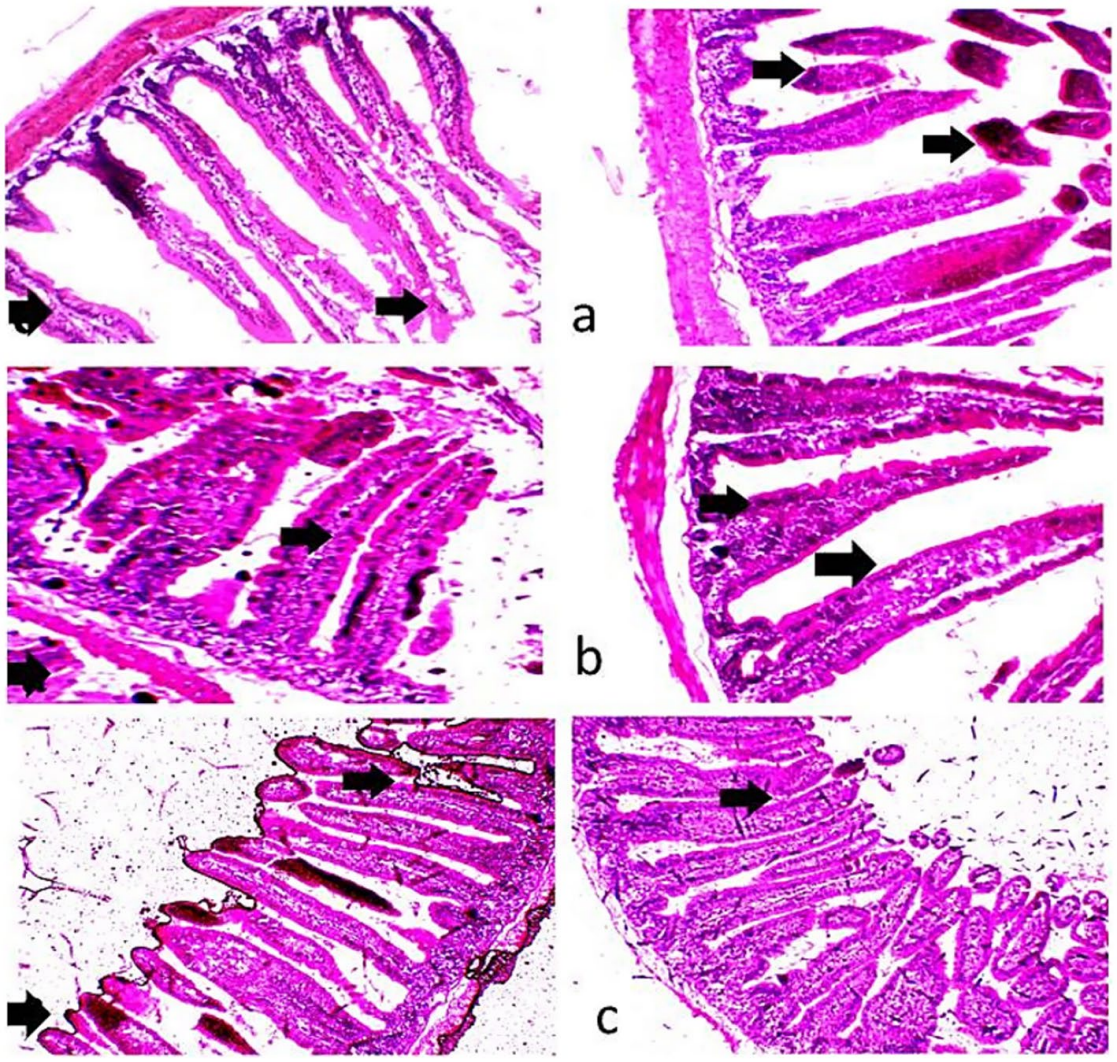
**(a–c)** Photomicrographs of the intestines of growing rabbits. The control group **(a)** exhibited a normal intestinal wall, while the 25% hydroponic barley replacement (CHB), and 25% hydroponic barley +0.5 g/kg enzymes (CHBE) groups **(b,c)** showed slight improvements in the architectural integrity of the mucosa and submucosa. (H&E, ×10).

Histological examination of the liver and ileum in the three dietary groups is shown in Figures 4, 5. The liver of rabbits in the CHB and CHBE groups displayed normally arranged hepatocytes and intact blood vessels, whereas the control group showed slight congestion and mild dilatation of blood vessels. In the ileum, the control group exhibited disruption of the intestinal wall, with desquamated and sloughed villi tips. In contrast, the CHB and CHBE groups showed improved ileal tissue architecture, with well-preserved mucosa and submucosa layers and properly arranged villi.

## 4 Discussion

The current study demonstrated that growing rabbits fed CHB and CHBE diets exhibited significantly higher BW and BW gain compared to the control group. This improvement in growth performance can likely be attributed to the superior nutritional quality and digestibility of the CHB and CHBE diets, which may enhance nutrient absorption and utilization, as supported by previous research (12). Moreover, rabbits on these diets showed the better feed conversion ratio relative to the control group, indicating more efficient use of feed for growth. This suggests that hydroponic barley, with or without enzyme supplementation, optimizes nutrient availability and metabolic efficiency, leading to improved overall growth and feed efficiency.

Hydroponic barley is known for its high fiber content, which is a critical aspect of its nutritional profile (Table 2). The fiber in hydroponic barley primarily consists of insoluble fibers such as cellulose and hemicellulose, which could benefit the digestive health of herbivorous animals like rabbits. This high fiber content supports gastrointestinal motility and promotes regular bowel movements, helping to maintain a healthy gut (17). Additionally, the water-soluble fibers in barley, such as beta-glucans, contribute to enhanced gut health, further supporting digestive health and potentially reducing the risk of gastrointestinal disorders common in rabbits, such as gastrointestinal stasis (24).

The digestible energy (DE) values provide critical context for interpreting intake effects. The minimal DE increases in CHB and CHBE groups (+1.1–1.3% vs. control) despite 4–5% higher intake suggest that HB’s liver/kidney metabolic improvements (Figure 3 and Table 4) reflect direct physiological effects rather than secondary energy surplus. Notably, the HB-alone group marked DE elevation (+6.1%) confirms its intrinsic energetic value, while the enzyme-supplemented groups’ patterns imply distinct nutrient-partitioning mechanisms. Future studies measuring fiber digestibility coefficients will help delineate these pathways.

Serum metabolic profiles serve as key indicators of an animal’s health, offering insights into its physiological, pathological, and nutritional state (13, 14). In this study, rabbits fed CHB and CHBE diets showed significantly lower serum ALT and AST levels, as well as reduced creatinine and urea concentrations compared to the control group. Additionally, serum cholesterol levels were significantly reduced in the CHB and CHBE groups. These findings suggest that hydroponic barley, with or without enzymes, did not impair liver or kidney function and may promote better metabolic health. Importantly, all these blood parameters stayed within normal levels, which is crucial, as elevated levels of AST, ALT, urea, and creatinine typically indicate liver and kidney dysfunction (1, 2, 13). The lower levels of these biomarkers in the CHB and CHBE groups align with previous studies, such as those by Mehrez et al. (11) and Shanti et al. (25), who observed similar results with hydroponic barley supplementation in rabbit diets.

While low serum cholesterol levels can be a concern in some species due to potential cardiovascular implications, the significance of this in rabbits is not well understood. In our study, the reduced cholesterol levels in the CHB and CHBE groups likely reflect a physiological response to the high-fiber content of the hydroponic barley, a characteristic known to influence lipid metabolism. High-fiber diets, such as those provided by hydroponic barley, can regulate lipid and glucose metabolism, potentially improving liver function and influencing blood lipid profiles. The observed cholesterol levels, although lower, remained within acceptable ranges and aligned with previous findings that high-fiber diets can help regulate metabolic parameters in rabbits (17, 26–30). Additionally, enzyme supplementation in our study seemed to enhance the metabolic profile further, supporting findings from other studies that report improvements in biochemical parameters, including reductions in cholesterol, triglycerides, AST, and ALT, in rabbits fed enzyme-supplemented, high-fiber diets (14). In terms of carcass characteristics, the inclusion of HB and HB with enzymes (CHBE) in the diet significantly improved the dressing percentage and reduced fat percentage compared to the control group. The dressing percentage, an indicator of meat yield, increased in the CHB and CHBE groups, while the reduction in fat percentage suggests that these diets help optimize lean meat production. These findings align with those of Nagadi (26), who observed similar improvements in carcass traits when rabbits were fed HB-based diets. Additionally, the longer cecum length observed in rabbits fed the CHBE diet may indicate improved nutrient absorption, likely due to the enzymatic enhancement of HB digestibility.

Despite these improvements in carcass and cecum characteristics, no significant differences were observed in the relative weights of vital organs such as the liver, heart, spleen, and kidneys. This suggests that the addition of HB, with or without enzymes, did not interfere with the normal development of these organs. These findings are consistent with prior research, which also reported no significant differences in organ weights between rabbits fed HB-based and control diets (9). However, other studies have shown mixed results, indicating that the impact of HB on organ development may vary depending on experimental conditions (31–33).

The gut microbiota plays a pivotal role in host health by modulating nutrient metabolism, immune responses, and resistance to pathogens (34). Our findings demonstrate that both CHB and CHBE diets significantly altered the cecal microbiota composition in rabbits, promoting a beneficial shift characterized by increased *Lactobacillus* spp. and reduced *E. coli* levels compared to the control group. Notably, the CHBE group exhibited the highest *Lactobacillus* spp. counts, suggesting that enzyme supplementation may further enhance the growth of these beneficial bacteria by improving fiber fermentability. This aligns with recent studies showing that enzyme-assisted fiber degradation increases the availability of fermentable substrates, selectively stimulating probiotic populations (35, 36). The rise in *Lactobacillus* spp. is particularly significant due to their well-documented role in gut health. These bacteria contribute to intestinal homeostasis through multiple mechanisms, including competitive exclusion of pathogens such as *E. coli* and *Salmonella* via bacteriocin production and niche competition (28). Additionally, *Lactobacillus* spp. enhances gut barrier function by producing short-chain fatty acids (SCFAs), which lower luminal pH and inhibit pathogenic colonization (37). The observed reduction in *E. coli* in both CHB and CHBE groups supports earlier findings that high-fiber diets suppress proteolytic bacteria while favoring saccharolytic fermentation (38). The further decline in *E. coli* with enzyme supplementation suggests that enhanced fiber breakdown may deprive pathogens of adhesion sites, reinforcing the diet’s protective effects (39). Our study is the first to report the effects of HB-based diets on rabbit gut microbiota, providing novel insights into how dietary fiber and enzyme supplementation influence microbial ecology. The synergistic benefits observed in the CHBE group are consistent with emerging research on *in vivo* multienzyme assemblies, which demonstrate improved fiber degradation and nutrient absorption in monogastric animals (40, 41). These findings highlight the potential of enzyme-fortified diets to optimize gut health while reducing reliance on antibiotics, a critical consideration for sustainable livestock production (42).

Histological examination of the liver and intestines revealed positive effects of the HB and CHBE diets. The livers of rabbits in these groups showed normal hepatocyte arrangement and intact blood vessels, while the control group exhibited mild congestion and slight dilation of hepatic blood vessels. Similarly, the intestinal mucosa and submucosa of rabbits fed CHB and CHBE diets maintained normal architecture, while the control group showed signs of villi sloughing and desquamation. These histological improvements suggest that HB, particularly when combined with enzymes, supports liver and intestinal health. *Lactobacillus* spp. is known to enhance the intestinal barrier by reinforcing tight junctions between intestinal cells, which could explain the improved gut integrity observed in the HB and CHBE groups. Furthermore, the high fiber content of HB promotes gastrointestinal motility, reduces the risk of constipation, and supports the growth of beneficial gut health, contributing to improved digestive health (43, 44). The effectiveness of enzyme supplementation in rabbits may vary depending on their age or life stage. Younger rabbits, with developing digestive systems, are likely to experience more pronounced benefits from enzyme supplementation, as their gastrointestinal tract is still maturing and may require additional support for optimal nutrient digestibility. In contrast, adult rabbits with more mature digestive systems may show less dramatic improvements, although they can still benefit from enhanced nutrient utilization. This variation underscores the need for further research to explore how age and life stage influence the effectiveness of enzyme supplementation to HB in rabbit nutrition. Future studies should investigate tailored enzyme supplementation strategies based on these factors. Additionally, while limited research has explored the effects of hydroponic barley (HB) with enzymes on internal organs and gut health in rabbits, our findings suggest that HB has the potential to influence both liver and intestinal health positively. To fully understand its potential, future research should focus on the impact of HB on gut microbiota composition and histological changes in internal organs, which would provide valuable insights into its role as a dietary supplement for improving rabbit health.

## 5 Conclusion

In conclusion, the present study demonstrates that incorporating hydroponic barley (HB), with or without enzymes, as 25% of the concentrated diet in growing rabbits significantly enhances their microbiological, physiological, histological, and carcass characteristics. HB shows great potential as a sustainable and innovative feed ingredient for improving rabbit health and productivity.

## Data Availability

The raw data supporting the conclusions of this article will be made available by the authors, without undue reservation.

## References

[1] Abdel-Wareth AAA Hammad S Ahmed H. Effects of *Khaya senegalensis* leaves on performance, carcass traits, hematological and biochemical parameters in rabbits. EXCLI J. (2014) 13:502–12. 26417277 PMC4463425

[2] Abdel-Wareth AAA Taha EMM Südekum K-H Lohakare J. Thyme oil inclusion levels in a rabbit ration: evaluation of productive performance, carcass criteria and meat quality under hot environmental conditions. Anim Nutr. (2018) 4:410–6. doi: 10.1016/j.aninu.2018.02.004, PMID: 30564761 PMC6284221

[3] Gupta JJ. Fodder production and livestock feeding management in eastern India (Unpub.). Patna, India: ICAR Research Complex for Eastern Region (2014).

[4] Naik PK Dhuri RB Swain BK Singh NP. Nutrient changes with the growth of hydroponics fodder maize. Indian J Anim Nutr. (2012) 29:161–3.

[5] Dung DD Godwin IR Nolan JV. Nutrient content and in sacco degradation of hydroponic barley sprouts grown using nutrient solution or tap water. J Anim Vet Adv. (2010) 9:2432–6. doi: 10.3923/javaa.2010.2432.2436

[6] Bakshi MPS Wadhwa M Makkar HPS. Hydroponic fodder production: a critical assessment. Broadening Horizons. (2017) 48:1–8. doi: 10.1079/PAVSNNR201712006, PMID: 40391582

[7] Jensen H Malter A. Protected agriculture: a global review. World Bank technical paper no. 253. Washington, DC: World Bank (1995). 156 p.

[8] Mooney J. Growing cattle feed hydroponically. Australia: Meat and Livestock (2005). 30 p.

[9] Abouelezz FM Hussein AM. Evaluation of baker's yeast (*Saccharomyces cerevisiae*) supplementation on the feeding value of hydroponic barley sprouts for growing rabbits. Egypt Poult Sci. (2017) 37:833–54. doi: 10.21608/epsj.2017.7735

[10] Gabr AA Mehrez AZ El-Ayek MY Gad AM. Effect of partial substitution of a commercial concentrate feed mixture crude protein by hydroponic barley fodder in diets of Apri rabbits on: 2-growth performance, slaughter and carcass traits, meat composition and economic efficiency. Egypt J Anim Prod. (2020) 57:121–6. doi: 10.21608/ejap.2020.100844

[11] Mehrez AZ Gabr AA El-Ayek MY Gad AM. Effect of partial substitution of a commercial feed crude protein by hydroponic barley fodder in diets of Apri rabbits on: 1-digestibility, feeding value, some blood constituents and caecum microflora count. J Anim Poult Prod. (2018) 9:453–8. doi: 10.21608/jappmu.2018.41160

[12] Abdel-Wareth AAA Mohamed EMH Hassan HA Eldeek AA Lohakare J. Effect of substituting hydroponic barley forage with or without enzymes on performance of growing rabbits. Sci Rep. (2023) 13:943. doi: 10.1038/s41598-023-27911-x, PMID: 36653392 PMC9849237

[13] Attia KA Saleh SY Abd El-Hamid S Zaki A Mohamed A. Effects of exogenous multi-enzyme feed additive (Kemzyme) on the activities of certain digestive enzymes and intestinal morphology in growing rabbits. J Agric Sci. (2011) 4:35–44.

[14] Abd El-Aziz AH El-Kasrawy NI Abo Ghanima MM Alsenosy AE-WA Raza SHA Khan S. Influence of multi-enzyme preparation supplemented with sodium butyrate on growth performance, blood profiles, and economic benefit of growing rabbits. J Anim Physiol Anim Nutr (Berl). (2020) 104:186–95. doi: 10.1111/jpn.13227 31657058

[15] Mathlouthi N Saulnier L Quemener B Larbier M. Xylanase, beta-glucanase, and other side enzymatic activities have greater effects on the viscosity of several feedstuffs than xylanase and beta-glucanase used alone or in combination. J Agric Food Chem. (2002) 50:5121–7. doi: 10.1021/jf011507b, PMID: 12188617

[16] Chudan S Kurakawa T Nishikawa M Nagai Y Tabuchi Y Ikushiro S. Beneficial effects of dietary Fiber in young barley leaf on gut microbiota and immunity in mice. Molecules. (2024) 29:1897. doi: 10.3390/molecules29081897, PMID: 38675716 PMC11054971

[17] Tian M Li D Ma C Feng Y Hu X Chen F. Barley leaf insoluble dietary Fiber alleviated dextran sulfate sodium-induced mice colitis by modulating gut microbiota. Nutrients. (2021) 13:846. doi: 10.3390/nu13030846, PMID: 33807544 PMC8001343

[18] Percie SN Ahluwalia A Alam S Avey MT Baker M Browne WJ. Reporting animal research: explanation and elaboration for the ARRIVE guidelines 2.0. PLoS Biol. (2020) 18:e3000411. doi: 10.1371/journal.pbio.3000411 32663221 PMC7360025

[19] De Blas C Mateos GG. Nutrition of the rabbit. 3rd ed. Wallingford, UK: CABI Publishing; (2020). p. 243–253.

[20] AOAC. Official methods of analysis. 17th ed. Arlington, VA: Assoc Off Anal Chem (2000).

[21] Salem WM Sayed WF Halawy SA Elamary RB. Physicochemical and microbiological characterization of cement kiln dust for potential reuse in wastewater treatment. Ecotoxicol Environ Saf. (2015) 119:155–61. doi: 10.1016/j.ecoenv.2015.05.012, PMID: 26004355

[22] SAS Institute. SAS/STAT® 9.2 user’s guide. 2nd ed. Cary, NC: SAS Institute Inc. (2009).

[23] Duncan DB. Multiple range and multiple F-tests. Biometrics. (1955) 11:1–42. doi: 10.2307/3001478

[24] Liu B Cui Y Ali Q Zhu X Li D Ma S. Gut microbiota modulate rabbit meat quality in response to dietary Fiber. Front Nutr. (2022) 9:849429. doi: 10.3389/fnut.2022.849429, PMID: 35392295 PMC8982513

[25] Shanti H Omar J Alwaheidi IN Abdallah J Dbadran E. Effect of substituting hydroponic barley for a commercial feed on performance and blood metabolites of growing Baladi rabbits. J New Sci. (2017) 39:2131–5.

[26] Nagadi SA. Replace the sprout barley instead of the concentrated fodder including anaerobic probiotic ZAD® for growing rabbits. Int J Eng Res Technol. (2019) 8:105–14. doi: 10.17577/IJERTV8IS100058

[27] Salama WA Refaie A El-Shora M. Performance of growing rabbits fed diets containing fennel seed meal without or with enzymes mixture. Egypt J Rabbit Sci. (2019) 29:45–60.

[28] El-Kelawy HM El-Kelawy MI. Impact of dietary supplementation with multi enzymes and/or probiotic on growth performance, nutrients digestibility and blood constituents of growing rabbits. Egypt J Nutr Feeds. (2016) 19:313–23. doi: 10.21608/ejnf.2016.74913

[29] El-Kelawy MI El-Shafey AS Hamdon HA. The effects of date stone meal with or without enzymes supplementation on growth performance, nutrient digestibility and economical efficiency of rabbits. Egypt J Nutr Feeds. (2020) 23:87–98. doi: 10.21608/ejnf.2020.95816

[30] El-Katcha MI Soltan MA El-Shiekh AI Ahmed HA Nomir MG. Influence of dietary enzymes supplementation on growth performance, nutrient digestibility, carcass traits and health status of growing rabbits. Alex J Vet Sci. (2013) 38:181–203.

[31] Attia KA Sohair YS Abd Elhamid SS Zaki AA Mohamed EA. Effects of exogenous multi-enzymes feed additive (Kemzyme) on the activities of certain digestive enzymes and intestinal morphology in growing rabbits. J Agric Sci. (2012) 4:35–44. doi: 10.5539/jas.v4n3p35

[32] Mohsen MK Abdel-Raouf EM Gaafar HM Yousif AM. Nutritional evaluation of sprouted barley grains on agricultural by-products on performance of growing New Zealand white rabbits. Nat Sci. (2015) 13:35–45.

[33] Morales M Fuente B Juárez M Ávila E. Short communication: effect of substituting hydroponic green barley forage for a commercial feed on performance of growing rabbits. World Rabbit Sci. (2009) 17:35–8. doi: 10.4995/wrs.2009.668

[34] Chen Y Shi Y Li M Ming D Liu W Xu X. Phase separation-mediated multienzyme assembly in vivo. J Agric Food Chem. (2025) 73:7867–76. doi: 10.1021/acs.jafc.4c09585, PMID: 40107849

[35] Wei X Wu H Wang Z Zhu J Wang W Wang J. Rumen-protected lysine supplementation improved amino acid balance, nitrogen utilization and altered hindgut microbiota of dairy cows. Anim Nutr. (2023) 15:320–31. doi: 10.1016/j.aninu.2023.08.001, PMID: 38053803 PMC10694044

[36] Yang X Xia X Zhang Z Nong B Zeng Y Wu Y. Identification of anthocyanin biosynthesis genes in rice pericarp using PCAMP. Plant Biotechnol J. (2019) 17:1700–2. doi: 10.1111/pbi.13133, PMID: 31004548 PMC6686123

[37] Dong L Dong F Guo P Li T Fang Y Dong Y. Gut microbiota as a new target for hyperuricemia: a perspective from natural plant products. Phytomedicine. (2025) 138:156402. doi: 10.1016/j.phymed.2025.156402, PMID: 39874797

[38] Zhang Y Zhang X Cao D Yang J Mao H Sun L. Integrated multi-omics reveals the relationship between growth performance, rumen microbes and metabolic status of Hu sheep with different residual feed intakes. Anim Nutr. (2024) 18:284–95. doi: 10.1016/j.aninu.2024.04.021, PMID: 39281047 PMC11402313

[39] Chen F Wang Y Wang K Chen J Jin K Peng K. Effects of *Litsea cubeba* essential oil on growth performance, blood antioxidation, immune function, apparent digestibility of nutrients, and fecal microflora of pigs. Front Pharmacol. (2023) 14:1166022. doi: 10.3389/fphar.2023.1166022, PMID: 37465523 PMC10350539

[40] Li H Zhou Y Liao L Tan H Li Y Li Z. Pharmacokinetics effects of *chuanxiong Rhizoma* on warfarin in pseudo germ-free rats. Front Pharmacol. (2023) 13:1022567. doi: 10.3389/fphar.2022.1022567, PMID: 36686675 PMC9849362

[41] Huang X Tang X Liao A Sun W Lei L Wu J. Application of cyclopropane with triangular stable structure in pesticides. J Mol Struct. (2025) 1326:141171. doi: 10.1016/j.molstruc.2024.141171

[42] Bai M Liu H Yan Y Duan S Szeto IM He J. Hydrolyzed protein formula improves the nutritional tolerance by increasing intestinal development and altering cecal microbiota in low-birth-weight piglets. Front Nutr. (2024) 11:1439110. doi: 10.3389/fnut.2024.1439110, PMID: 39555191 PMC11565607

[43] Fasiullah MS Khandaker ZH Islam KM Kamruzzaman M Islam R. Effect of dietary enzymes supplementation on nutrient utilization and growth performance of rabbit. Int J Biol Res. (2010) 1:17–21.

[44] Hong KJ Lee CH Kim SW. Aspergillus oryzae GB-107 fermentation improves nutritional quality of food soybeans and feed soybean meals. J Med Food. (2004) 7:430–5. doi: 10.1089/jmf.2004.7.430, PMID: 15671685

[45] Fekete S. The new Hungarian system for evaluation of feed energy. In Proceeding of 1st north American rabbit congress, October, 10–13, Portland or, USA (1987)

